# The cingulate island sign within early Alzheimer’s disease-specific hypoperfusion volumes of interest is useful for differentiating Alzheimer’s disease from dementia with Lewy bodies

**DOI:** 10.1186/s13550-016-0224-5

**Published:** 2016-09-13

**Authors:** Etsuko Imabayashi, Kota Yokoyama, Tadashi Tsukamoto, Daichi Sone, Kaoru Sumida, Yukio Kimura, Noriko Sato, Miho Murata, Hiroshi Matsuda

**Affiliations:** 1 Integrative Brain Imaging Center, National Center of Neurology and Psychiatry, 4-1-1 Ogawahigashi, 187-8551 Kodaira, Tokyo Japan; 2 Department of Radiology, National Center for Global Health and Medicine, 1-21-1 Toyama, 162-8655 Shinjuku, Tokyo Japan; 3 Department of Radiology, National Center of Neurology and Psychiatry, 4-1-1 Ogawahigashi, 187-8551 Kodaira, Tokyo Japan; 4 Department of Radiology, The University of Tokyo, 7-3-1 Hongo, 113-8654 Bunkyoku, Tokyo Japan; 5 Department of Neurology, National Center of Neurology and Psychiatry, 4-1-1 Ogawahigashi, 187-8551 Kodaira, Tokyo Japan

**Keywords:** Brain perfusion SPECT, CIS, DLB, Imaging, Alzheimer’s disease

## Abstract

**Background:**

In addition to occipital hypoperfusion, preserved metabolism of the posterior cingulate gyri (PCG) relative to the precunei is known as the cingulate island sign (CIS) in the patients with dementia with Lewy bodies (DLB). CIS has been detected using [^18^F]fluorodeoxyglucose positron emission tomography but not using brain perfusion single-photon emission computed tomography (SPECT). The purpose of this study was to optimize brain perfusion SPECT to enable differentiation of DLB from Alzheimer’s disease (AD) using CIS and occipital hypoperfusion.

Eighteen patients with probable DLB and 17 age-matched Pittsburgh compound B-positive patients with AD underwent technetium-99m ethyl cysteinate dimer SPECT. SPECT *Z*-score maps were generated using the easy *Z*-score imaging system (eZIS) analysis software (Matsuda H, Mizumura S, Nagao T, Ota T, Iizuka T, Nemoto K, Takemura N, Arai H, Homma A, AJNR Am J Neuroradiol 28(4):731–6, 2007), which included volumes of interest (VOIs) in which a group comparison between patients with AD and cognitively normal subjects revealed significant relative hypoperfusion. We used the Montreal Neurological Institute (MNI) space anatomical border to divide the bilateral PCG to precunei VOIs into two parts, the PCG and precunei. *Z*-scores in the PCG, precunei, and occipital areas and ratios were analysed and compared with receiver operating characteristic (ROC) curve analyses.

**Results:**

The largest area under the curve (AUC) value for use in differentiating DLB from AD with the ratio of PCG to medial occipital was 0.87; the accuracy, sensitivity, and specificity were 85.7, 88.9, and 82.4 %, respectively. The AUC with the ratio of PCG to the precuneus was smaller, and it was 0.85, though no significant difference was observed between these two AUCs.

**Conclusions:**

The *Z*-score ratio of the PCG within the early-AD-specific VOI to medial-occipital area is clinically useful in discriminating demented patients with DLB from those with AD.

**Electronic supplementary material:**

The online version of this article (doi:10.1186/s13550-016-0224-5) contains supplementary material, which is available to authorized users.

## Background

Dementia with Lewy bodies (DLB) is one of the most frequent causes of dementia in elderly people, with Alzheimer’s disease (AD) being the most prevalent cause. It is clinically important to distinguish DLB from AD because specific side effects of antipsychotic drugs are limited to DLB. In discriminating DLB from AD clinically, dopamine transporter (DAT) imaging [1] and [^123^I]metaiodobenzylguanidine (MIBG) myocardial scintigraphy [2] are useful because they detect early disturbances of the nigrostriatum or peripheral sympathetic nervous system in patients with DLB. Using a combination of these two techniques, Shimizu et al. [3] reported over 90 % sensitivity and specificity in discriminating DLB from AD. However, brain perfusion single-photon emission computed tomography (SPECT) is more widely and commonly used for clinical screening and examination of patients with dementia. Compared with morphometric imaging, SPECT is a more sensitive modality for functional imaging used to detect the early stages of neurodegenerative disease before shrinkage [4]. SPECT also reveals useful information for differentiating AD as well as other dementias, including vascular dementia or frontotemporal lobe degeneration [5].

In patients with AD, proportional hypometabolism and hypoperfusion are commonly observed in temporoparietal regions in general and in the posterior cingulate gyri (PCG) in particular. Perfusion decreases observed in patients with DLB overlap with those observed in patients with AD; however, more occipital hypometabolism and hypoperfusion are reportedly observed in patients with DLB [6, 7]. Conversely, Kemp et al. [8] reported that only 28 % of hexamethylpropyleneamine oxime (HMPAO) SPECT scans of patients with DLB revealed occipital hypoperfusion.

In 1997, Imamura et al. [9] reported the relative preservation of cingulate glucose metabolism in patients with DLB compared with those having AD. In this study, brain perfusion SPECT analysis was optimized for differentiation of AD from DLB. Lim et al. [10] found that preservation of glucose metabolism in the mid- or posterior cingulate, known as the cingulate island sign (CIS), is highly specific for diagnosing DLB. They also reported that the sensitivities ranged from 62 to 86 % for the CIS and from 43 to 50 % for the medial occipital lobe. Graff-Radford et al. [11] recently reported the results from an investigation using [^18^F]fluorodeoxyglucose positron emission tomography ([^18^F]FDG-PET) imaging and the pathologic association of CIS with autopsy and carbon 11-labelled Pittsburgh compound B ([^11^C]PiB) PET imaging in patients with DLB. They concluded that the CIS was indicative of the lower Braak neurofibrillary tangle (NFT) stage in patients with DLB.

In contrast, O’Brien et al. [12] stated that the accuracy of differentiating AD from DLB was disappointingly poor and not yet at the level of being clinically useful. They also claimed that brain perfusion SPECT imaging did not reveal the CIS in patients with DLB. Therefore, in this study, the entire precunei and PCG anatomical area was not included and the CIS within the early-AD-specific volumes of interest (VOIs) [13] was analysed. We aimed to determine the usefulness of a *Z*-score map of technetium-99m ethyl cysteinate dimer ([^99m^Tc]ECD) brain perfusion SPECT images for discriminating patients with DLB from those with AD. Brain perfusion SPECT is not only more commonly used but is also a more economical modality compared with [^18^F]FDG-PET.

## Methods

### Patients

This was a retrospective study that used data obtained at a single medical centre.

All the subjects with AD were included in previously reported paper from our institute [14]. This previous study was approved by the National Center of Neurology and Psychiatry Ethics Committee for Clinical Research, and informed consent was obtained from all the subjects. For our study, public notification was applied according to the requirement of the ethical committee. Then, the ethical committee approved our retrospective study using preliminary obtained images.

At first, 20 of the PiB-positive patients who fulfil the criteria of probable AD with a high level of evidence of the AD pathophysiological process, with both atrophy of the medial temporal lobe and decrease of the brain perfusion by over two standard deviations based on the *Z*-scores, were observed as described in this paper [14]. The [^11^C]PiB amyloid PET results were confirmed by two board-certified nuclear medicine physicians. After reinvestigation of clinical data and MRI, three patients were excluded: one who was first diagnosed as DLB, another had infarction in the brain stem on the T2-weighted image, and the other had complication of brain amyloid angiopathy. The average lag time between PET and SPECT acquisition was 1.40 ± 2.33 (mean ± SD) months.

For DLB, from chart screening, among all accessible patients who underwent brain perfusion SPECT, those fulfil the probable DLB on the basis of the criteria proposed in the third consortium on DLB international workshop [15] were selected. Finally, 18 patients with probable DLB (M/F = 10:8; age, 73.9 ± 6.8 years) and 17 patients with AD (M/F = 6:11; age, 73.6 ± 8.9 years) were studied.

### Brain perfusion SPECT

In advance, all patients received an intravenous line and an intravenous injection of 740 MBq [^99m^Tc]ECD (Fujifilm RI Pharma, Tokyo, Japan) was administered while lying down with eyes closed in dark, quiet surroundings. The global CBF was noninvasively measured using graphic analysis as described previously [16–18], without blood sampling. The passage of tracer from the aortic arch to the brain was monitored for 100 s at 1-s intervals. Regions of interest (ROIs) were hand-drawn over the aortic arch (ROI_aorta_) and both brain hemispheres (ROI_brain_). A hemispheric brain perfusion index (BPI) [16] was determined before the start of the initial back diffusion of the tracer from the brain to the blood as follows:

1 $$ \mathsf{B}\mathsf{P}\mathsf{I}=\mathsf{100}\times \mathsf{k}\mathsf{u}\frac{\mathsf{10}\times {\mathsf{ROI}}_{\mathsf{aorta}}\mathsf{size}}{{\mathsf{ROI}}_{\mathsf{brain}}\mathsf{size}}, $$

where ku is the unidirectional influx rate for the tracer from the blood to the brain, determined by the slope of the line in graphic analysis within the first 30 s after injection. Then, BPI (*x*) was converted to global CBF values (*y*) obtained by ^133^Xe inhalation SPECT studies (*y* = 2.60*x* + 19.8) [16].

Ten minutes later, SPECT imaging was performed on a two-head gamma camera and six-slice CT system (Symbia T6; Siemens, Erlangen, Germany) equipped with low-energy, high-resolution, and parallel-hole collimators. Ninety views were obtained continuously throughout 360° of rotation (4°/step, 128 × 128 matrix, zoom 1.45). The voxel size was 3.3 × 3.3 × 3.3 mm. To reconstruct the SPECT image, a combination of Fourier rebinning followed by ordered subset expectation maximization (iteration number 8 and subset 10) and a 7-mm full width at half maximum Gaussian filter was used. Attenuation correction was performed using the CT data and Chang’s method [19]. CT attenuation-corrected images were used for measuring global CBF, and Chang’s attenuation-corrected images were used for *Z*-score analysis because the database included in the software was reconstructed using Chang’s attenuation-correction methods. To calculate CBF and to correct for incomplete retention of [^99m^Tc]ECD in the brain, the following linearization algorithm [20] of a curve-linear relationship between the brain activity and blood flow was applied:

2 $$ \mathsf{F}\mathsf{i}=\mathsf{F}\mathsf{r}\times \frac{\mathit{\mathsf{a}}\times \left(\raisebox{1ex}{$\mathsf{C}\mathsf{i}$}\!\left/ \!\raisebox{-1ex}{$\mathsf{C}\mathsf{r}$}\right.\right)}{\left[\mathsf{1}+\mathit{\mathsf{a}}-\left(\raisebox{1ex}{$\mathsf{C}\mathsf{i}$}\!\left/ \!\raisebox{-1ex}{$\mathsf{C}\mathsf{r}$}\right.\right)\right]}, $$

where Fi and Fr represent CBF values for a region *I* and a reference region, respectively, and Ci and Cr are the SPECT counts for the region *i* and the reference region, respectively. The cerebral hemisphere was used as the reference region, and global CBF obtained from the graphic analysis was substituted for Fr. The linearization factor *a* was set to 2.59, which was a proposed value by Friberg et al. [20].

### Image preprocessing

*Z*-score maps of the obtained SPECT images were converted using the easy *Z*-score imaging system (eZIS) analysis software (Fujifilm RI Pharma Co., Ltd., Tokyo, Japan). It included spatial normalization parameters in statistical parametric mapping (SPM)2 (http://www.fil.ion.ucl.ac.uk/spm/) and a [^99m^Tc]ECD brain template in the same space as the Montreal Neurological Institute (MNI) standard brain template. Normal databases are included in this software, and inter-institutional differences can be corrected. That is, correction can be made for data obtained in the institute where the database was built, using previously scanned Hoffman 3-D Brain Phantom™ data.

**Fig. 1:**
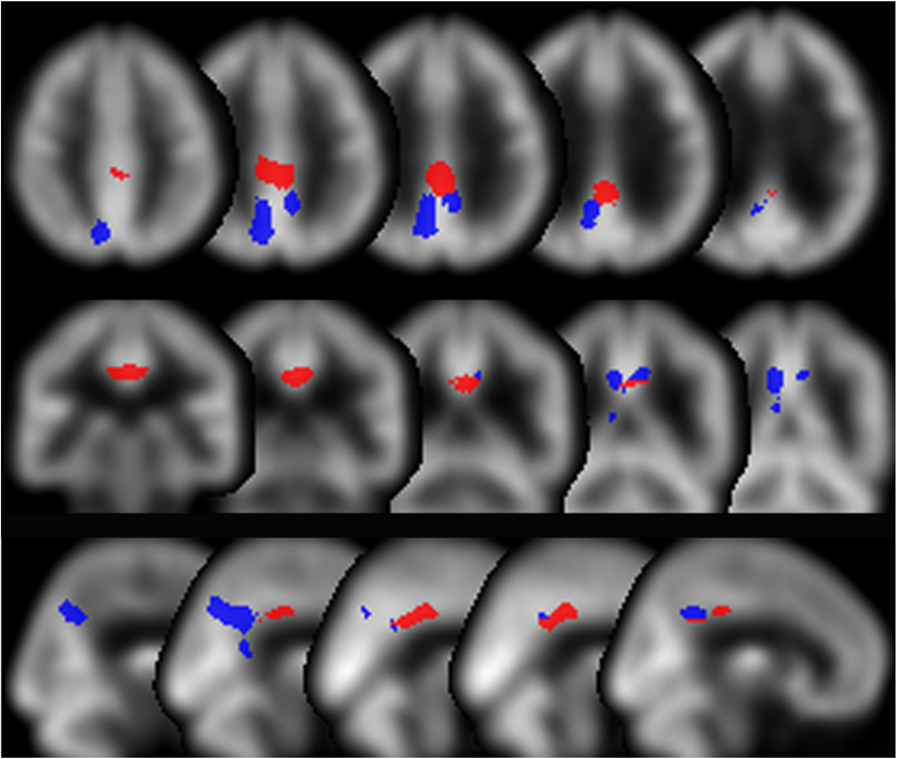
Volumes of interest (VOIs). The *areas* are the parts of the early Alzheimer’s disease (AD)-specific VOIs in the easy *Z*-score imaging system (eZIS) located in the posterior cingulate to the precunei area. *Blue area*: Precuneus_AD_VOIs which are the areas of overlap of the precuneus VOIs in the Automated Anatomical Labeling (AAL) atlas and the early-AD-specific VOIs. *Red area*: Posterior_Cingulate_AD_VOIs which are the areas of overlap of the cingulum VOIs in the AAL atlas and the early-AD-specific VOIs

After the inter-institutional correction, specially normalized [^99m^Tc]ECD SPECT images from each patient were compared with normal images from the age-matched database: ECD60-69y DB and ECD70y DB, using voxel-by-voxel *Z*-score analysis after pixel normalization to the global mean values [*Z*-score = ([control mean] − [individual value])/(control SD)] as previously reported by Minoshima et al. [21]. The VOIs in areas of significant perfusion reduction were included in this software; these areas were identified in patients with AD following a group comparison with cognitively healthy individuals [22]. Among these preset VOIs, we chose VOIs that were set within the bilateral posterior cingulate to the precunei area; we then used the border between the Cingulum_VOIs and the Precuneus_VOIs in the Automated Anatomical Labeling (AAL) atlas to split the VOIs into two parts, the bilateral PCG_AD_VOIs and the Precuneus_AD_VOIs (Fig. 1). We also assembled VOIs in the medial and lateral occipital areas in this atlas and constructed the Medial_Occipital_VOI and the Lateral_Occipital_VOI. Additionally, we constructed the Whole_Occipital_VOI as the sum of these two VOIs. Then, we summated the positive *Z*-scores within each VOI.

For the CIS, we first divided each value: the sum of all the positive *Z*-scores in the PCG_AD_VOI by that in the Precuneus_AD_VOI and named it as CISpreC. Second, in order to compare the value of cingulate preservation to the value of occipital hypoperfusion, we divided the PCG_AD_VOI by the Medial_Occipital_VOI, the Lateral_Occipital_VOI, or the Whole_Occipital_VOI. We named their three values as CISmedO, CISlatO, and CISwO; CISmedO = PCG_AD_VOI/Medial_Occipital_VOI, CISlatO = PCG_AD_VOI/Lateral_Occipital_VOI, and CISwO = PCG_AD_VOI/Whole_Occipital_VOI.

The area under the receiver operating characteristic (ROC) curve (AUC) was obtained by thresholding each of these values for all VOIs, CISpreC, CISmedO, CISlatO, and CISwO. Finally, the AUCs were statistically compared [23] (Table 2).

**Fig. 2:**
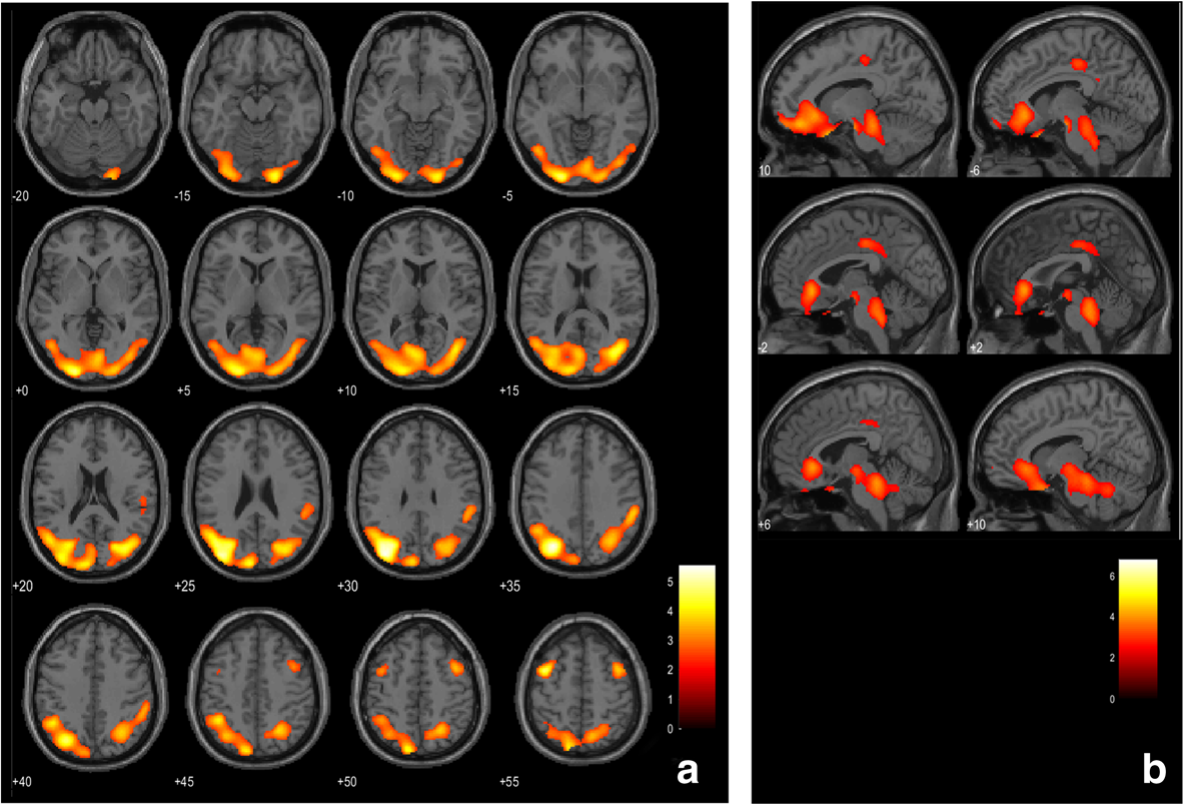
Significance maps (*uncorrected p < 0.01, with extent threshold k=400 voxels)* superimposed on a T1-weighted brain MRI template image in the Montreal Neurological Institute (MNI) space. The *colour bar* represents the *t* value. **a** Significance maps of proportionally decreased brain perfusion in patients with DLB comparing with patients with AD. **b** Significance maps of proportionally increased brain perfusion in patients with DLB comparing with patients with AD. *AD* Alzheimer’s disease, *DLB* dementia with Lewy bodies, *MRI* magnetic resonance imaging

We also added group comparison between AD and DLB subjects using SPM2. We added the results as Additional file 1 (Fig. 2). VOIs used for analyses were integrated in the images.

## Results

The patient characteristics are summarized in Table 1. No significant difference was observed for age and gender. The disease duration was longer in patients with DLB. The mean cerebral blood flow of the whole brain was significantly lower in patients with DLB. All the heart-to-mediastrum ratios in [^123^I]MIBG scanned 3 h after injection in patients with DLB were below 2.0.

**Table 1:** Demographic data

	AD	DLB
Age at the time of SPECT	74.4 ± 8.5	73.9 ± 6.8
Male/female	5/12	10/8
Disease duration at the time of SPECT (years)	2.0 ± 1.6	4.0 ± 2.3**
MMSE	21.5 ± 3.3	19.1 ± 6.7
Cerebral blood flow (mean; mL/100 g/min)	39.79 ± 3.22	36.86 ± 4.16*
Heart-to-mediastrum ratio in [^123^I]MIBG (3 h after injection)	NA	1.33 ± 0.14 (range 1.09–1.62)

*AD* Alzheimer’s disease, *DLB* dementia with Lewy bodies, *SPECT* single-photon emission computed tomography, *MMSE* Mini-Mental State Examination, *[* ^*123*^ *I]MIBG* [^123^I]metaiodobenzylguanidine, *NA* not applicable

*p = 0.0200; **p = 0.0027

The AUCs and the statistical results are shown in Table 2. The greatest AUC value was 0.873 when the ratio was the sum of all the positive *Z*-scores within the PCG_AD_VOIs to those in the Medial_Occipital_VOIs; CISmedO were used as thresholds. The accuracy, sensitivity, and specificity were 85.7, 88.9, and 82.4 %, respectively, for differentiating patients with DLB from those with AD. According to the AUC comparison, the AUC calculated using CISpreC was smaller but not significantly different from the AUC thresholded with CISmedO; the accuracy, sensitivity, and specificity were 80.0, 66.7, and 94.1 %, respectively.

**Table 2:** Area under the receiver operating characteristic (ROC) curve (AUC)

VOIs	AUC ± SE	95 % CI	*p* ^a^
CISpreC	0.850 ± 0.068	0.688 to 0.947	
CISmedO	0.873 ± 0.064	0.716 to 0.961	*0.589*
CISlatO	0.771 ± 0.081	0.598 to 0.896	*0.271*
CISwO	0.820 ± 0.074	0.654 to 0.929	*0.559*
Precuneus_AD_VOI	0.618 ± 0.098	0.438 to 0.776	0.016
PCG_AD_VOI	0.716 ± 0.092	0.538 to 0.855	*0.078*
Medial_Occipital_VOI	0.614 ± 0.102	0.435 to 0.773	0.032
Lateral_Occipital_VOI	0.585 ± 0.100	0.407 to 0.748	0.021
Whole_Occipital_VOI	0.631 ± 0.098	0.451 to 0.787	0.029

*VOI* volume of interest, *SE* standard error, *CI* confidence interval, *PCG* posterior cingulate gyri, *CISmedO* PCG_AD_VOI/Medial_Occipital_VOI, *CISlatO* PCG_AD_VOI/Lateral_Occipital_VOI, *CISwO* PCG_AD_VOI/Whole_Occipital_VOI

^a^The AUCs of the ROC curves were compared to those thresholded with CISpreC [23]. The italicized *p* values were not significantly different from the AUC thresholded with CISpreC

**Fig. 3:**
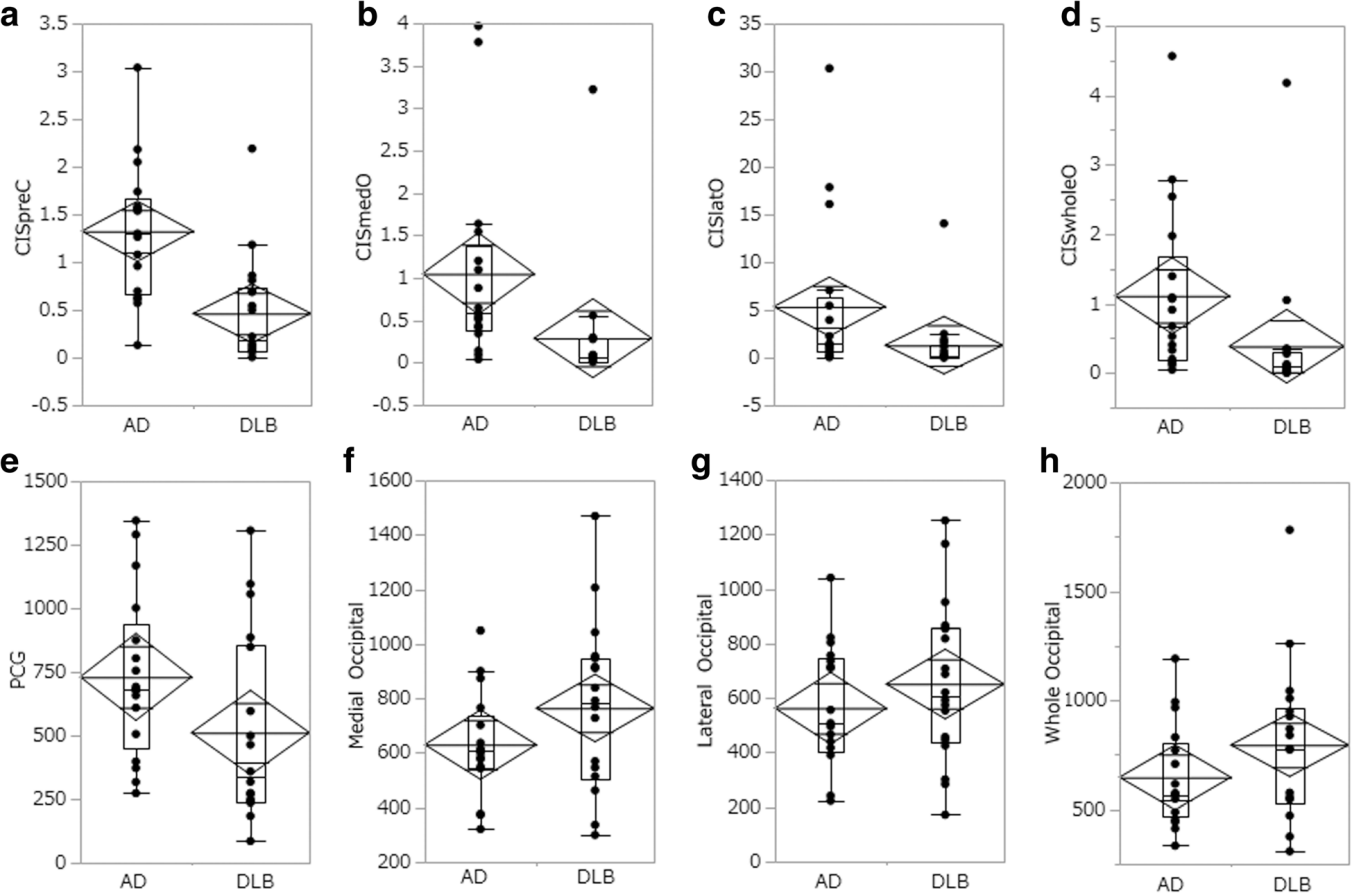
Box plots of CISpreC, CISmedO, CISlatO, CISwO, PCG_AD_VOI, and three occipital VOIs. **a** CISpreC. **b** CISmedO, **c** CISlatO, **d** CISwO, **e** Average positive *Z*-scores in the PCG_AD_VOI, **f** Average positive *Z*-score in the Medial_Occipital_VOIs. **g** Average positive *Z*-score in the Lateral_Occipital_VOIs. **h** Average positive *Z*-score in the Whole_Occipital_VOIs. The *rhombus* shows the mean and confidential interval. The *boxes* show the upper and lower quantiles with the median, and the *whiskers* show the minimum, maximum, or 1.5 * interquartile range

The AUCs thresholded with occipital VOIs were significantly smaller than the AUCs thresholded with CISpreC. Box plots of the CISpreC and the three occipital VOIs are shown in Fig. 3.

## Discussion

Lim et al. [10] reported that the accuracy of differentiating AD from DLB using the ratio of the posterior cingulate to the surrounding cortex was 78 % with visual inspection of [^18^F]FDG-PET images. They found reduced glucose metabolism in the lateral occipital area but preserved glucose metabolism in the mid- or posterior cingulate; this phenomenon, known as CIS, is specific to DLB.

A previous study [24] showed that the most significant differences in the region of interest (ROI) analysis of brain perfusion images between the AD and DLB groups were in the occipital area and the precuneus. Conversely, another study [8] reported that a diagnosis of DLB could not be ruled out in using occipital hypoperfusion. Therefore, in our study, we compared the *Z*-score of the occipital area and the CIS to differentiate patients with DLB from those with AD. We found that the *Z*-score ratio of the posterior cingulate to the precuneus was superior to the occipital *Z*-score for the purpose of discriminating DLB from AD.

The demographic data showed that patients with DLB had a lower cerebral blood flow value compared with that found in patients with AD. This result is consistent with that from a previous report [25]. Fong et al. [26] reported that this hypoperfusion may be a potential indicator of cholinergic dysfunction. They observed improvements in both hypoperfusion and cognitive impairments with arterial spin-labelled perfusion magnetic resonance imaging (MRI) after loading a single dose of cholinesterase inhibitor. In the early stage, quantitative cerebral blood flow might be used as a referential parameter to differentiate AD from DLB; however, we cannot eliminate other reasons for the induction of hypoperfusion. Our results suggested that the CIS could be more useful as a specific indicator to differentiate AD from DLB using brain perfusion SPECT.

Recently, Graff-Radford et al. [11] reported results of a comparison of the CIS in [^18^F]FDG-PET and [^11^C]PiB-PET scans. They also investigated autopsy results using the CIS. They concluded that the CIS was indicative of a lower Braak NFT stage in patients with DLB; however, it was not associated with fibrillary β-amyloid deposition. In our study, we compared the CIS detected using brain perfusion SPECT in patients with DLB and AD. We cannot compare these two studies directly because they did not describe the discrimination accuracy. The greater overlap observed in the box plots from our SPECT study suggested that the CIS observed using [^18^F]FDG-PET might more accurately discriminate patients with DLB from those with AD. Brain perfusion and metabolism are physiologically coupled [27]. The limitations of SPECT include lower image resolution and a large partial volume effect. These limitations in conjunction with a patient’s pathological status result in decreased accuracy when attempting to differentiate between DLB and AD using brain perfusion SPECT. However, SPECT is widely accessible and more economical.

O’Brien et al. [12] found that the accuracy of differentiating AD from DLB was disappointingly poor and not at a level where it is likely to be clinically useful. They showed that, even with FDG, the differentiation accuracy was 72.4 %. Further, the CIS was not observed in the SPECT images in their study. In our study, the high accuracy (80.0 %) was obtained when a CISpreC value of 0.54 was used for thresholding. We used the software-provided VOIs obtained from a group comparison between patients with AD and cognitively normal subjects. We divided these VOIs into the precuneus area and the posterior cingulate area along the anatomical border. Because AD and DLB are known to have pathological overlap and it is difficult to distinguish these two diseases using [^18^F]FDG-PET or brain perfusion SPECT [12], it is logical to use these early-AD-specific VOIs to discriminate AD from DLB.

Though all patients with AD underwent [^11^C]PiB amyloid PET, none of the patients with DLB did; this was due to the retrospective nature of the investigation, which was based on clinical diagnoses. The percentage of PiB-positive scans in patients with DLB ranged between 30 and 85 % [28]. Generally, high rates of positive amyloid scans are consistent with the high pathological frequency of amyloid plaques; thus, it is unlikely that amyloid PET would be helpful in differentiating DLB from AD in our study.

In discriminating DLB from AD clinically, Shimizu et al. [3] reported over 90 % sensitivity and specificity using DAT imaging [1] and [^123^I]MIBG myocardial scintigraphy [2]. With these images, early disturbances of the nigrostriatum or peripheral sympathetic nervous system in patients with DLB can be detected directly more accurately comparing with our results. However, brain perfusion single-photon emission computed tomography (SPECT) is more widely and commonly used for clinical screening and examination of patients with dementia. SPECT reveals useful information for differentiating AD as well as other dementias, including vascular dementia or frontotemporal lobe degeneration [5]. Compared with morphometric imaging, SPECT is a more sensitive modality for functional imaging used to detect the early stages of neurodegenerative disease before shrinkage in AD [4].

O’Brien et al. [12] claimed that brain perfusion SPECT imaging did not reveal the CIS in patients with DLB. Although, in this study, obtained AUC was almost equal to O’Brien’s results, those obtained from ROI analysis with [^18^F]FDG-PET. It is speculated that when our procedure is applied to [^18^F]FDG-PET images, AD and DLB might be more accurately differentiated. But brain perfusion SPECT is not only more commonly used but is also a more economical modality compared with [^18^F]FDG-PET.

## Conclusions

Using the CIS within the early-AD-specific VOI, DLB and AD were differentiated with 80 % accuracy. This accuracy was superior to the differentiation ability of the medial occipital VOI analysis. Although differentiation of DLB from AD is difficult with visual inspection [12], our findings indicate that the *Z*-score ratio of the posterior cingulate and the precuneus within the early-AD-specific VOI is clinically useful in discriminating demented patients with DLB from those with AD.

## Additional file

Additional file 1: Tables S1–S2. Areas where proportionally significant decrease perfusion was found in patients with DLB comparing to patients with AD. (DOCX 14 kb)
