# Supplementary material for: The cingulate island sign within early Alzheimer’s disease-specific hypoperfusion volumes of interest is useful for differentiating Alzheimer’s disease from dementia with Lewy bodies
Source: EJNMMI Res. 2016 Sep 13;6(1):67. doi: 10.1186/s13550-016-0224-5 (PMC5020033; doi:10.1186/s13550-016-0224-5)
Supplement: Additional file 1: Tables S1–S2. — Areas where proportionally significant decrease perfusion was found in patients with DLB comparing to patients with AD. (DOCX 14 kb) [file 13550_2016_224_MOESM1_ESM.docx]

**Table 3** Areas where proportionally significant decrease perfusion was found in patients with DLB comparing to patients with AD

|  | Brodmann | Cluster size | Peak *p* | Peak | Talairach coordinate | | |
| --- | --- | --- | --- | --- | --- | --- | --- |
|  | area | voxels | (uncorrected) | *t* vaslue | (x, y, z) | | |
| L parieto-occipital area | 39 | 19072 | 0.000 | 5.55 | -30 | -70 | 32 |
| L middle frontal cortex | 6 | 455 | 0.000 | 4.78 | -38 | 9 | 53 |
| R middle frontal cortex | 8 | 420 | 0.000 | 3.89 | 40 | 11 | 51 |

**Table 4** Areas where proportionally significant dincrease perfusion was found in patients with DLB comparing to patients with AD

|  | Brodmann | Cluster size | Peak *p* | Peak | Talairach coordinate | | |
| --- | --- | --- | --- | --- | --- | --- | --- |
|  | area | voxels | (uncorrected) | *t* vaslue | (x, y, z) | | |
| L orbitofrontal cortex | 11 | 16232 | 0.000 | 6.8 | -16 | 42 | -19 |
| R cerebellum |  | 474 | 0.000 | 3.69 | 27 | -89 | -28 |
| L posterior cingulate | 31 | 447 | 0.001 | 3.13 | -3 | -22 | 39 |
